# Baseline soil-transmitted helminth and schistosome infection in the Geshiyaro project, Ethiopia: A unique transmission interruption project using biometric fingerprinting for longitudinal individual analysis

**DOI:** 10.1371/journal.pntd.0011589

**Published:** 2023-10-18

**Authors:** Anna E. Phillips, Alison K. Ower, Kalkidan Mekete, Ewnetu Firdawek Liyew, Rosie Maddren, Birhan Mengistu, Ufaysa Anjulo, Melkie Chernet, Julia C. Dunn, Hussein Mohammed, Habtamu Belay, Bokretsion Gidey, Geremew Tasew, Gemechu Tadesse, Mihretab Salasibew, Getachew Tollera, Roy Anderson

**Affiliations:** 1 MRC Centre for Global Infectious Disease Analysis, Imperial College London, St Mary’s Campus, London, United Kingdom; 2 London Centre for Neglected Tropical Disease Research, London, United Kingdom; 3 Ethiopian Public Health Institute, Addis Ababa, Ethiopia; 4 Children’s Investment Fund Foundation, London, United Kingdom; Consejo Nacional de Investigaciones Cientificas y Tecnicas, Fundación Mundo Sano, ARGENTINA

## Abstract

**Background:**

The Geshiyaro project aims to assess the feasibility of interrupting transmission of soil-transmitted helminths (STH) and schistosome (SCH) infection in the Wolaita zone of southern Ethiopia through high coverage community-wide mass drug administration (MDA), in combination with improved water, sanitation, and hygiene services and behaviour change communication delivered through the existing health care infrastructure. To accurately measure treatment coverage a population census was conducted enrolling individuals with biometric fingerprinting and barcoded ID cards. This paper details the baseline census and parasitology surveys conducted before the start of any interventions.

**Methods:**

The census was conducted in five of the 15 Wolaita districts between October 2018 and December 2019, enrolling all consenting participants from every household. Simultaneously, a cross-sectional parasitology survey was conducted in 130 out of 361 randomly selected communities from all 15 districts, with 100 individuals across all age groups (infant to adult) per community providing stool and urine for analysis by duplicate Kato-Katz and a point-of-care circulating cathodic antigen (POC-CCA) to test for *Schistosoma mansoni* and STH, and microhaematuria and urine filtration for *Schistosoma haematobium*. Of the 130 communities, 30 were randomly selected for annual, longitudinal parasitological monitoring, with 150 randomly selected individuals from infant to adult providing two days of stool and urine samples for analysis by the same diagnostic tests per community.

**Results:**

In total 97,919 households participated in the baseline census enrolling 466,071 individuals, with parasitological data obtained from 10,785 people. At baseline, 15.5% were infected with at least one STH species, with *Ascaris lumbricoides* (9.5%), followed by hookworm (7.2%) and *Trichuris trichiura* (1.8%). Substantial heterogeneity in STH prevalence was observed between communities ranging from 0% to 61% where most infections were low intensity. *Schistosoma mansoni* infection was the dominant schistosome infection (0.85% by Kato-Katz and 13.3% by POC-CCA trace negative and 21.5% trace positive), with few *Schistosoma haematobium* infections identified (2.77% haematuria positive and 0.13% positive by urine filtration).

**Conclusions:**

While the national control program in Ethiopia has made good progress in reducing prevalence of STH and SCH in Wolaita since it was launched in 2015, there remain areas of persistent infection suggesting the existence of environmental or behavioural risk factors that contribute to ongoing transmission. This project aims to identify the most efficient intervention strategies to reduce community burden and reach interruption of transmission.

## Author summary

The standard method of control for intestinal worms, a common public health problem in sub-Saharan Africa, is distribution of deworming treatment on a mass scale. These infections are difficult to eliminate as re-infection is common due a combination of people not being treated and high exposure to the parasites through poor access to sanitation and clean water. The Geshiyaro project in Ethiopia has the ambitious goal of breaking the transmission cycle of such infections through treatment in combination with provision of water and sanitation services. The impact of these interventions is being measured using a unique approach, that is, using finger printing to evaluate prevalence of infection with compliance to treatment and access water or sanitation.

## Introduction

Helminth infections caused by soil-transmitted helminths (STH) and schistosome parasites (SCH) predominantly affect those living in poverty in low and middle income countries, with an estimated 1.5 billion people (2016) and 2.3 billion people (2019) thought to be infected, respectively [1]. While STH and SCH are unlikely to result in mortality, they contribute to significant morbidity including malnutrition, impaired physical and cognitive development, and anaemia [2,3]. In sub-Saharan Africa, the predominant species of STH and SCH infecting humans are *Ascaris lumbricoides*, *Trichuris trichiura*, hookworms (*Necator americanus*, *Ancylostoma duodenale*) and *Schistosoma mansoni* and *Schistosoma haematobium*.

The primary method of controlling these infections is through repeated mass drug administration (MDA) whereby a population, typically school-age children (SAC), are treated irrespective of infection status [4]. This is a cost-effective way of controlling the intensity of infection and morbidity, but re-infection often occurs due to the failure of helminth infections to illicit strong acquired immunity to reinfection. Concurrently, the lack of improved sanitary conditions enables continued transmission in endemic communities [5]. Over the last two decades, drug donations by GlaxoSmithKline (Albendazole), Merck (Praziquantel) and Johnson & Johnson (Mebendazole) have been instrumental in the substantial reductions in STH and SCH prevalence achieved largely through school-based deworming [6]. There is growing concern about the future of these donations, however, as national control programs move from morbidity control to elimination, which requires increased community drug coverage [7, 8]. Mathematical modelling suggests that interruption of transmission may be feasible if treatment is expanded to include adults (STH and SCH) and pre-SAC (STH only) with high treatment coverage [8–10]. Without this increase in targeted population, interruption of transmission may not be achieved, and de-worming may be required in some communities indefinitely. The necessary MDA coverage in pre-SAC and adults is dependent on the magnitude of the basic reproductive number, R_0_, which describes transmission intensity in a defined setting [10].

Several trials have investigated the impact of alternative treatment strategies to the standard school-based MDA on the feasibility of breaking STH and SCH transmission. The TUMIKIA trial in Kenya demonstrated a greater reduction in STH prevalence, predominantly hookworm, with annual and biannual community-wide treatment (CWT) compared to the standard annual school-based deworming (SBD) [11]. Similarly, the SCORE study in Niger demonstrated that biannual CWT was more impactful on reducing SCH when starting prevalence is high [12]. The DeWorm3 randomised control trial in India, Benin, Malawi is also currently assessing the impact of biannual CWT on STH [13].

Recent mathematical modelling has illustrated the potential added benefit of water, sanitation and hygiene (WaSH) interventions alongside preventive chemotherapy (PC) to sustain the progress achieved through deworming [14]. Given the SCH transmission cycle, requiring water contact with water contaminated with the cercarial stage of the parasite released from the intermediate snail host [15–16], and *A*. *lumbricoides* and *T*. *trichiura* acquired through the ingestion of helminth eggs via the faecal oral route, it follows that improvements to WaSH infrastructure with strong community acceptance and utilisation should complement deworming [4]. There has, however, been mixed evidence to link the benefits of WaSH interventions on STH and SCH infection, which may be due to insufficient duration of studies, heterogeneity in uptake, difficulty in measuring impact of WaSH concomitant to deworming, and individual association with WaSH being considered rather than community-wide benefits to improved coverage [17,19].

Ethiopia has one of the highest burdens of helminth infections, with an estimated 53.3 million people at risk for SCH and 96.7 million at risk for STH [21]. In 2013, disease mapping was conducted to inform the strategy for the national implementation of PC for SCH and STH infections. MDA campaigns in schools began shortly thereafter, and in 2015 the Federal Ministry of Health launched its National Deworming Program, with the ambitious aim to interrupt transmission by 2030 [20].

The Geshiyaro project is a multi-year study located in the Wolaita zone of the Southern Nations, Nationalities, and People’s Region (SNNPR) in southwestern Ethiopia whose primary aim is to determine the feasibility of interrupting transmission of STH and SCH through high coverage CWT complemented by WaSH and behaviour change communication (BCC) [21]. This proof of concept study is implemented through the existing infrastructure of the Federal Ministry of Health. In this paper, we describe the data collected through the Geshiyaro census, the first of which has used biometric enrolment in the context of NTDs, performed in five districts, in addition to baseline epidemiological patterns of STH and SCH across all 15 districts in Wolaita prior to the initiation of CWT and WaSH interventions delivered through Geshiyaro.

## Methods

### Ethics statement

Ethical approval has been obtained from the Institutional Review Board (IRB) at the Scientific and Ethical Review Office of the Ethiopian Public Health Institute. In census areas, consent was given using biometric enrolment. In non-census areas, verbal informed consent was obtained from study participants (by adults in the case of children under 16 years) after providing an informative overview of the aim and procedure of the study in the local language, which was documented electronically through the data collection form. Data analysis was performed without names and biometric data was converted to numeric IDs for the sake of confidentiality. All participants were aware of their right to refuse to provide information, as well as the ability to drop out of the study after consent. All participants were made aware that future treatment would not be contingent on census registration. All individuals identified as infected with STH or SCH were treated at the subsequent MDA with either PZQ 40 mg/kg or ALB respectively.

### Study design

The Geshiyaro protocol has been published [21]. In brief, the aim of Geshiyaro is to evaluate the most efficient intervention strategies to reduce endemic communities with low prevalence to elimination of transmission. The project has three intervention arms: Arm 1, expanded CWT, with improved WaSH and BCC; Arm 2, expanded CWT alongside the One WaSH National Programme; and Arm 3 (control), the national SBD and One WaSH National Programme. The Wolaita zone of SNNPR, southwestern Ethiopia, is split between Arm 1 (five districts) and Arm 2 (ten districts). Arm 3 sites are in districts bordering of Wolaita.

The project consists of three phases: Phase 1 (2018/2019) a pilot year in one district, largely to assess the feasibility of the conducting a census with a biometric; Phase 2 (originally 2019–2023 but now extended to 2025 due to COVID-19) where activities were rolled out in all 15 districts of Wolaita; and Phase 3 (2026/2027) a two-year observation period to confirm transmission interruption. This paper presents baseline data collected in Phase 1 and the first year of Phase 2.

### Study area

**Fig 1:**
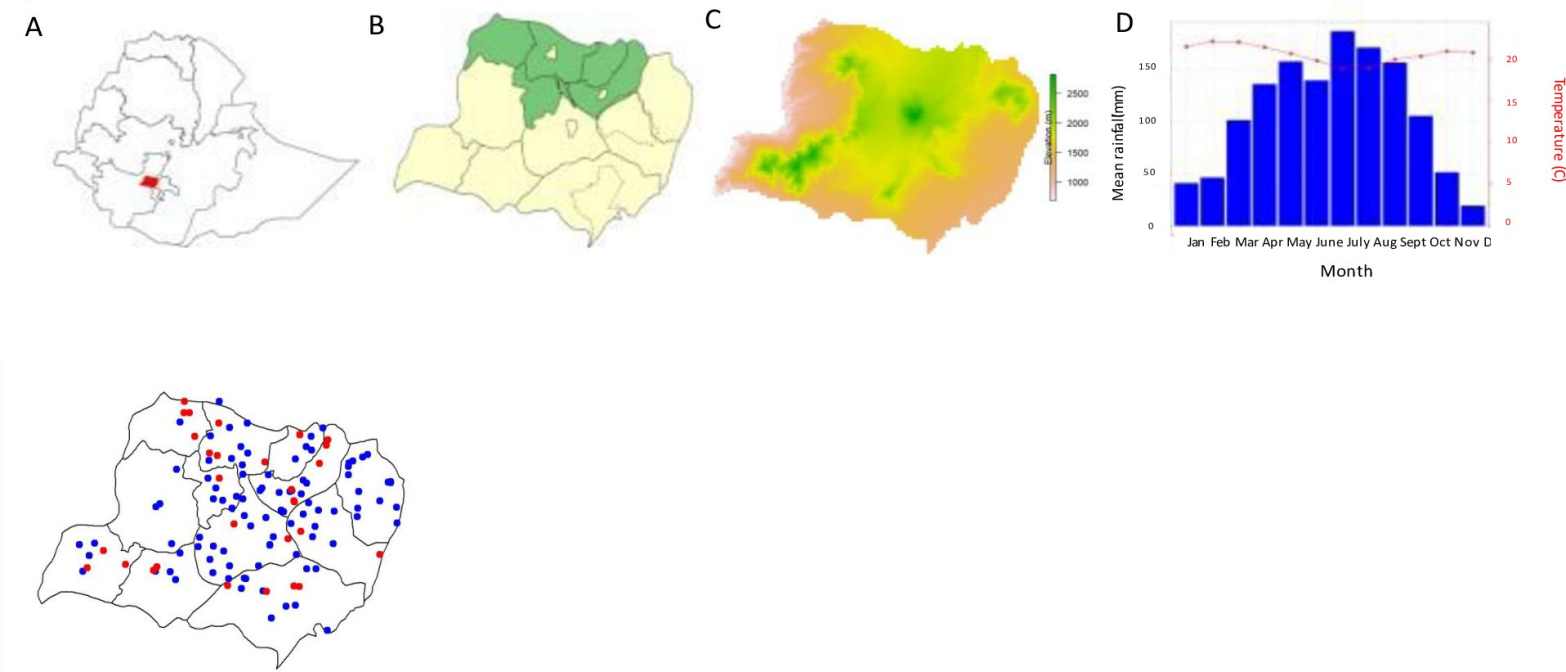
**A—Geshiyaro Study Site.** Location of Wolaita (red) in SNNPR, Ethiopia (a); Allocation of districts into study arms, Arm 1 (green) expanded, community-wide MDA and enhanced WASH, Arm 2 (yellow) expanded, community-wide MDA with National OneWASH programme only; elevation in metres across Wolaita (c); and average rainfall and temperature across Wolaita by month (d); and **B**—**Baseline mapping (blue) and combined mapping/annual sentinel sites (red)** This map was created in R software program and the source of the basemap within the package used to generate the map was Global Administrative Areas (2012). GADM database of Global Administrative Areas, version 2.0. [online] URL: www.gadm.org.

At the start of the project in 2018, the Wolaita zone was composed of 15 districts (Arms 1 and 2). In 2019, the zone was redistricted into 22 districts whereby one of the five census districts, Abela Abaya, was created from the larger Humbo district (dotted lines in Fig 1A). The majority of Wolaita is rural, with three main peri-urban/urban districts Sodo, Areka, and Boditi Towns.

### Census

Initially, a pilot district (Bolosso Sore, Arm 1) was conducted in October-December 2018 to assess the acceptability and feasibility of conducting a biometric census. As a result of lessons learned in the first year, including the amount of sensitisation and data cleaning required to acquire a final denominator, the household census was then scaled back to five out of 15 districts due to financial and logistical constraints. In Year 2, four additional districts (Bolosso Bombe and Damot Gale, Arm 1 and Damot Weydie and Abela Abaya, Arm 2) conducted a biometric census in October-December 2019. The objective of the census was to provide an accurate and up-to-date estimate of the population eligible for treatment. The household survey included a detailed questionnaire on WaSH infrastructure and practices. All data was collected electronically using Android smartphones using SurveyCTO (Dobility, Inc; Cambridge, MA, USA).

**Fig 2:**
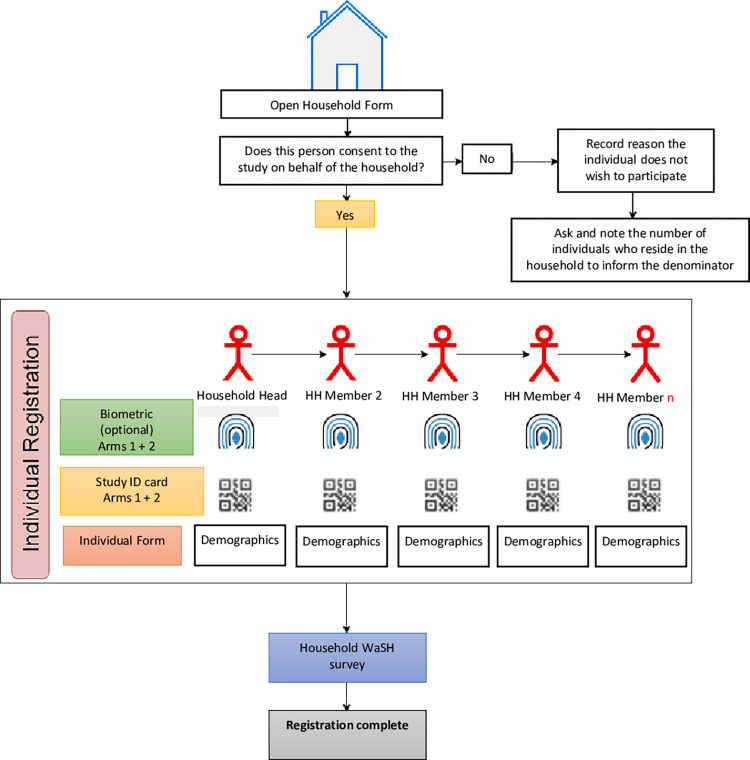
Household data collection workflow. Workflow of enrolment in the census including individual registration by biometric and/or study ID card followed by collection of individual demographic information and lastly a household WASH survey.

Each village in Ethiopia is split into subunits (or Gotts) whereby the households are typically numbered and correspond to an individual health folder at the village health post. When the team of surveyors visited the village, each surveyor was randomly allocated a gott. To enrol each household, the surveyors walked in a sequential fashion within each gott to ensure all were enumerated and then verify against the family folders to ensure no households were missed. All households within the five census districts were enrolled, with all individuals who regularly resided within the household (e.g. not visiting family for a short-period of time) eligible for inclusion in the census count, irrespective of their presence at the time of survey. Consent for enrolment of the household and all its members was provided by the household head or any other adult household member (defined as ≥ 21 years old). All household members consenting to the population census aged ≥2 years were registered with a study ID card, and for those that accepted, also with a biometric fingerprint. The household data collection workflow is shown in Fig 2. Biometric fingerprints were captured using a Vero fingerprint scanner and Android smartphone app developed by Simprints (Cambridge, UK). These unique identifiers enable the longitudinal linkage of a participant’s census registration, including household WaSH data, with their subsequent individual treatment record at each round of MDA and parasitology results (subject to inclusion in a parasitology survey) [19]. Enumerators returned up to three times to capture biometrics from those who were not previously present. All household heads were informed that access to MDA would not be contingent on participation in the census. In September 2019, a census mop-up was performed in the pilot district Bolosso Sore (one year after the census) to increase biometric registration and register any households previously missed or declined.

### Baseline parasitological mapping: Study sites and participants

A cross-sectional baseline parasitology mapping survey was conducted in 130 villages randomly selected across the 15 districts from 361 eligible villages with 10,785 individual records retained for analysis after data cleaning from October-December 2018. In each village 100 individuals were surveyed, stratified equally into five age groups: pre-SAC (0–4 years), SAC (5–14 years), adolescents (15–20 years), young adults (21–35 years), and adults (36+ years), evenly distributed by gender. Individuals were selected using family folders at the village health post with a sampling interval (i.e., for a village of 1,000 households every 10th folder was selected for a total of 100 households). The family folder lists the name and age of each family member at each household, which enabled selection across all age groups and sex. The census was not used to select individuals for the parasitological mapping to help verify anyone missed in the census. If the selected individual or entire household had been missed in the census, they were then registered separately. If a household had been censused, individuals were identified using their study ID card and/or biometric to later link their parasitology results with the household WaSH data and MDA coverage.

In the census villages, an individual consented using their biometric/study ID card. In non-census villages, consent for biological (stool and urine) samples was obtained from the individual (age ≥16 years) provided from the parent/guardian and child (age <16 years). Each consenting mapping participant then provided a single stool and urine sample on the day of collection. Stool samples were assessed by duplicate Kato Katz (KK) for STH *(A*. *lumbricoides*, *T*. *trichiura*, hookworm) and *S*. *mansoni* infection [22]. Slides were read within one hour of preparation on the day of sample collection. Given the low prevalence of *S*. *mansoni*, a more sensitive point-of-care circulating cathodic antigen (POC-CCA, Rapid Medical Diagnostics, Pretoria, South Africa) was also tested on each urine sample. A urine dipstick (Haemastix: Siemens Healthcare Diagnostics GmbH, Eschborn, Germany) was used to assess presence of microhaematuria, as a proxy of *S*. *haematobium* [23]. Since haematuria is well documented good proxy for *S*. *haematobium* infection, only haematuria positive urine samples (trace haemolysed, +, ++, and +++ were considered positive) were filtered for quantitative egg counts of *S*. *haematobium* [23].

### Baseline sentinel sites: Study sites and participants

Thirty sentinel sites were randomly selected from the 130 mapping villages, stratified by low, moderate, and high prevalence STH and SCH baseline results, to provide longitudinal impact monitoring at each site (see Fig 1B for location of mapping and sentinel sites). Phase I (pilot survey in one district) baseline sentinel sites were conducted in January 2019 (four sites in Bolosso Sore) and Phase II (in the remaining 14 districts) baseline sentinel sites were conducted in February-March 2020 (26 sites). In each of the 30 sentinel sites, 150 individuals were enrolled into the cohort to allow for loss-to-follow due to the longitudinal nature of the cohort, stratified by age and sex (15 male and female participants in each of the five age groups described above), using their biometric and/or Geshiyaro study ID card. All participants in the sentinel site cohort were enrolled independent to the mapping survey but using the same sampling method of family folders in the health post with a sampling interval. In total 4,401 individuals were enrolled across the 30 sentinel sites. Some individuals were enrolled in both the baseline parasitology mapping and sentinel sites. Since prevalence and intensity was expected to decrease over the period of the cohort, an additional day of data collection was conducted in the sentinel sites where participants provided a single urine sample and two separate stool samples over consecutive days. The same diagnostic methods were used the baseline mapping, only differing by the total number of Kato Katz slides (two slides per individual in the mapping and four slides for sentinel sites). The age-sex stratification of participants in the two surveys is shown in S1 Fig.

### Data analysis

All data and statistical analyses and figure generation were performed in RStudio (R version 3.6.0, Vienna, Austria). Data analysis was performed on the baseline census survey from all five districts and the census mop-up in the pilot district (Bolosso Sore). Household and population figures are described using numbers, percentages/proportions, and arithmetic means. Data cleaning entailed numerous internal and external consistency checks including construction of population pyramids of age to identify any irregularities in the reported data where age-sex ratio scores were compared between the Geshiyaro census, national census, US Bureau of Census and Survey population, and district housing census counts. The sample size calculations for Geshiyaro have been published elsewhere [21].

Prevalence estimates include all samples whereby for some individuals this would be one sample (mapping) and for others this could be the average of two samples (sentinel sites), with 95% confidence intervals calculated using the Clopper-Pearson method [24]. A single outlier was removed from the analysis due to an exceedingly high *A*. *lumbricoides* intensity, 9,694,296 eggs per gram (epg), which was likely a counting or data entry error. Prevalence and intensity estimates are presented for both baseline mapping and sentinel site data (S1 and S2 Tables). Associations between categorical variables (e.g. study arm, sex) and binary outcome variable (e.g. STH/SCH prevalence) were analysed using Chi-squared tests. The level of statistical significance was set at p<0.05. Infection intensity was defined using the WHO intensity thresholds into light, moderate, and heavy intensity infections [25]. Confidence intervals for intensity values were determined using parametric mean epg adjusted percentiles (95% two-sided, bias-corrected and accelerated—BCa) given the negative binomial distribution of the raw data and calculated using bootstrapping with the “boot” package [26].

### WaSH definitions

A separate paper has been published on the associations found between WaSH access and the prevalence of STH and SCH found at baseline in the Geshiyaro project [19]. Definitions for household WaSH status are in line with the World Health Organization and United Nations Children’s Fund (WHO/UNICEF) Joint Monitoring Programme for Water Supply, Sanitation and Hygiene (JMP) guidelines [27]. In brief, JMP defines “basic water” source is access to water from an improved drinking source (piped water connection into dwelling; public tap or standpipe; tube well or borehole; protected dug well; protected spring; and/or rainwater collection) that is reliable and accessible within a 30 minutes round trip of the household (including queuing time); a “limited source” is drinking water from an improved source where collection time exceeds 30 minutes for a round trip; “unimproved” drinking water is from an unprotected dug well or unprotected spring; and “no service” includes drinking water collected from surface water including a river, dam, lake, pond, stream, canal or irrigation canal. “Basic sanitation” is defined as access to an improved facility (these include flush or pour-flush toilets to piped sewer systems, septic tanks, pit latrines, VIP latrines, pit latrine with slab, or composting toilets) that is not shared with another household; “limited sanitation” as improved facilities shared between two or more households; “unimproved sanitation” as the use of a pit latrine without a slab or a bucket latrine. Exposure to surface water, a risk factor for SCH, was defined as collecting drinking or cooking/handwashing water, washing clothes and/or bathing in/with water collected from a river, stream, lake.

### Parameter estimates

The level of aggregation of parasite burden per person was measured using the surrogate marker of epg of stool or ml of urine, measured inversely by the negative binomial aggregation parameter. The aggregation parameter (k) is estimated using the formula below, relating k with the mean egg counts and prevalence of infection. Here, the likelihood of counting a number of KK positive individuals from the sampled pool of individuals follows a positive binomial distribution, and the distribution of egg counts from KK slides follows a negative binomial, giving the relationship between prevalence (P) and mean intensity (M_egg_). The loglikelihood was computed in R using the dbinom package.


$$P=1-{\left(1+\frac{Megg}{k}\right)}^{-k}$$

## Results

### Census

In total 466,071 individuals were enrolled across five censused districts, of which 426,044 (91.4%) registered by study ID card and 254,506 (54.6%) consented to biometric registration, across 97,919 households. The proportion of households consenting to census varied by district from 84.4% in Damot Weydie up to 95.5% in Bolosso Bombe and Abela Abaya, Table 1. In comparison to the World Bank population estimates, young men and women in their early twenties and pre-school age children were relatively under sampled, S2 Fig.

**Table 1:** Population enrolled in the Geshiyaro census (September-December 2018 and August 2019, Bolosso Sore; October-December 2019; Bolosso Bombe, Damot Gale, Damot Weydie, Abela Abaya).

	Bolosso Sore	Bolosso Bombe	Damot Gale	Damot Weydie	Abela Abaya
Study Arm	Pilot/Arm 1	Arm 1	Arm 1	Arm 2	Arm 2
Size (km^2^)	303.5	272.3	242.0	215.8	204.9
n Villages (sites)	32	20	29	24	16
n Households (HH)	33377	16278	21413	18180	8671
Household Consent Rate (%)	93.41	95.47	87.39	84.44	95.49
Total Population^1^	170248	77370	101662	75216	41575
Registered Population^2^	158980	74008	89278	64100	39678
Biometric Registered Population	93876	44562	53936	38859	23273
% Population pre-SAC	12.91	13.21	12.23	11.88	13.96
% Population SAC	32.71	34.44	30.85	29.61	33.70
% Population Adult (15-20y)	15.86	13.69	15.02	14.41	14.03
% Population Adult (21-35y)	22.63	22.49	21.67	23.36	23.12
% Population Adult (36+y)	15.90	16.17	20.22	20.74	15.19
					

^1^The ‘Total Population’ refers to the estimated total population composed of the registered population and the estimated population declining census registration.

^2^Registered population refers to the population consenting to census who were registered with biometric and/or study ID card.

The mean number of people per household was 4.8, with little variation between districts. The majority of household dwellings are owned, with clay/mud walls, earth floors, a man-made roof (e.g. iron sheet) and electricity was limited (<25%), except for Abela Abaya district.

Access to safely managed/basic drinking water was greatest in Bolosso Sore district (43.92% basic) and lowest in Damot Weydie (22.58% basic), Table 2. Most households had unimproved sanitation facilities and access to basic sanitation was low (<20%). Open defecation was practised in all districts (defined by absence of access to a latrine) and highest in Abela Abaya (7.72%). Exposure of household members to surface water was high in all districts (>50%).

**Table 2:** Characteristics of households enrolled in the Geshiyaro census by district.

	Bolosso Sore	Bolosso Bombe	Damot Gale	Damot Weydie	Abela Abaya
***Mean People per Household***	5.1	4.8	4.8	4.2	4.8
***Own Dwelling*** %	98.2	95.9	99.0	95.1	96.8
***Dwelling Walls***—Man Made (%)	0.5	0.7	0.3	1.1	0.8
Clay/Mud (%)	80.0	87.5	87.4	89.4	84.9
Wood (%)	19.5	11.8	12.3	9.5	14.2
***Dwelling Floors***—Man Made (%)	2.30	4.6	0.9	5.0	4.0
Wood (%)	0.7	0.6	0.3	0.6	0.1
Earth (%)	97.0	94.8	98.9	94.4	95.9
***Dwelling Roof***—Man Made (%)	76.1	83.1	89.2	90.9	70.0
Grass/Thatch (%)	22.8	16.0	10.4	8.8	29.9
Mud (%)	1.1	0.9	0.5	0.3	0.1
***Electricity*** %	17.5	14.3	14.0	21.4	55.3
***Drinking Water***					
Basic (%)	43.92	34.16	33.08	22.58	35.85
Limited (%)	26.91	29.56	39.54	42.34	44.36
Unimproved (%)	17.12	20.25	13.84	19.95	9.07
No Service (%)	8.04	15.14	12.27	8.37	9.06
***Basic sanitation***					
Basic (%)	8.73	5.30	5.76	4.91	5.41
Limited (%)	4.02	2.98	1.04	1.84	0.92
Unimproved (%)	62.83	75.95	75.16	81.06	63.96
Open Defecation (%)	15.04	10.47	10.26	7.72	24.55
***Exposure to Surface Water***	77.64	82.20	65.10	68.30	56.80

### Baseline parasitology

#### Prevalence of STH Infection

Overall prevalence of at least one STH species was 15.52%, with significant heterogeneity (0–61.0%), Table 3. *A*. *lumbricoides* was the most prevalent STH infection at 9.47%, followed by hookworm (7.24%) and *T*. *trichiura* (1.78%). Prevalence of moderate intensity infections was low across all species: 7.54% of *A*. *lumbricoide*s infections, 18.96% *T*. *trichiura*, and 0.55% hookworm. Only a single heavy intensity STH infection was observed (*T*. *trichiura*, 10,800 epg).

**Table 3:** Prevalence and intensity of infection (baseline mapping and sentinel sites).

Species	n Positive	Prevalence (%)	95% CI	Village Prevalence Range (%)^*1*^	Mean Intensity (epg)	n Moderate/Heavy Infection (%)^*2*^
Any STH	2348	15.52	14.94–16.1	0–61		
*A*. *lumbricoides*	1433	9.47	9.01–9.95	0–46.8	131.18	108 (7.54%)
*T*. *trichiura*	269	1.78	1.57–2	0–15.4	13.37	51 (18.96%)
Hookworm	1095	7.24	6.83–7.66	0–53.2	11.73	6 (0.55%)
*S*. *mansoni* (KK)	129	0.85	0.71–1.01	0–12.3	0.76	11 (8.53%)
*S*. *mansoni* (POC trace-)	1867	13.31	12.75–13.88	0–44.9		
*S*. *mansoni* (POC trace+)	3251	21.55	20.89–22.21	0–54		
Haemastix Positive	411	2.77	2.51–3.05	0–14.7		
*S*. *haematobium*^*3*^	20	0.13	0.08–0.21		0.46	0

^1^For the kebele prevalence range, kebeles must have > 4 datapoints for the diagnostic to be included in the range

^2^% of infection positive individuals

^3^*S*. *haematobium* infections were determined by presence of eggs following urine filtration. The prevalence of *S*. *haematobium* infection refers to the number of *S*. *haematobium* egg-positive individuals within all individuals who provided urine samples, as urine filtration was only performed on Haemastix positive samples

There was a difference in prevalence between study arms of any STH in Arm 1 as 23.10% (CI 22.01–24.26%) compared to 11.17% (CI 10.55–11.82%) in Arm 2 (*χ*^2^ = 380.3, p <0.0001). *A*. *lumbricoides* prevalence was 16.81% (CI 15.83–17.83%) in Arm 1 compared to 5.27% (CI 4.84–5.74%) in Arm 2 (*χ*^2^ = 542.4, p <0.0001) and *T*. *trichiura* prevalence was 3.00% (CI 2.56–3.48%) in Arm 1 compared to 1.08% (CI 0.88–1.31%) in Arm 2 (*χ*^2^ = 72.8, p <0.0001). Hookworm prevalence was slightly higher in Arm 1 at 8.03% (CI 7.33–8.78%) compared to 6.78% (CI 6.29–7.30%) in Arm 2 (*χ*^2^ = 8, p = 0.005).

**Fig 3:**
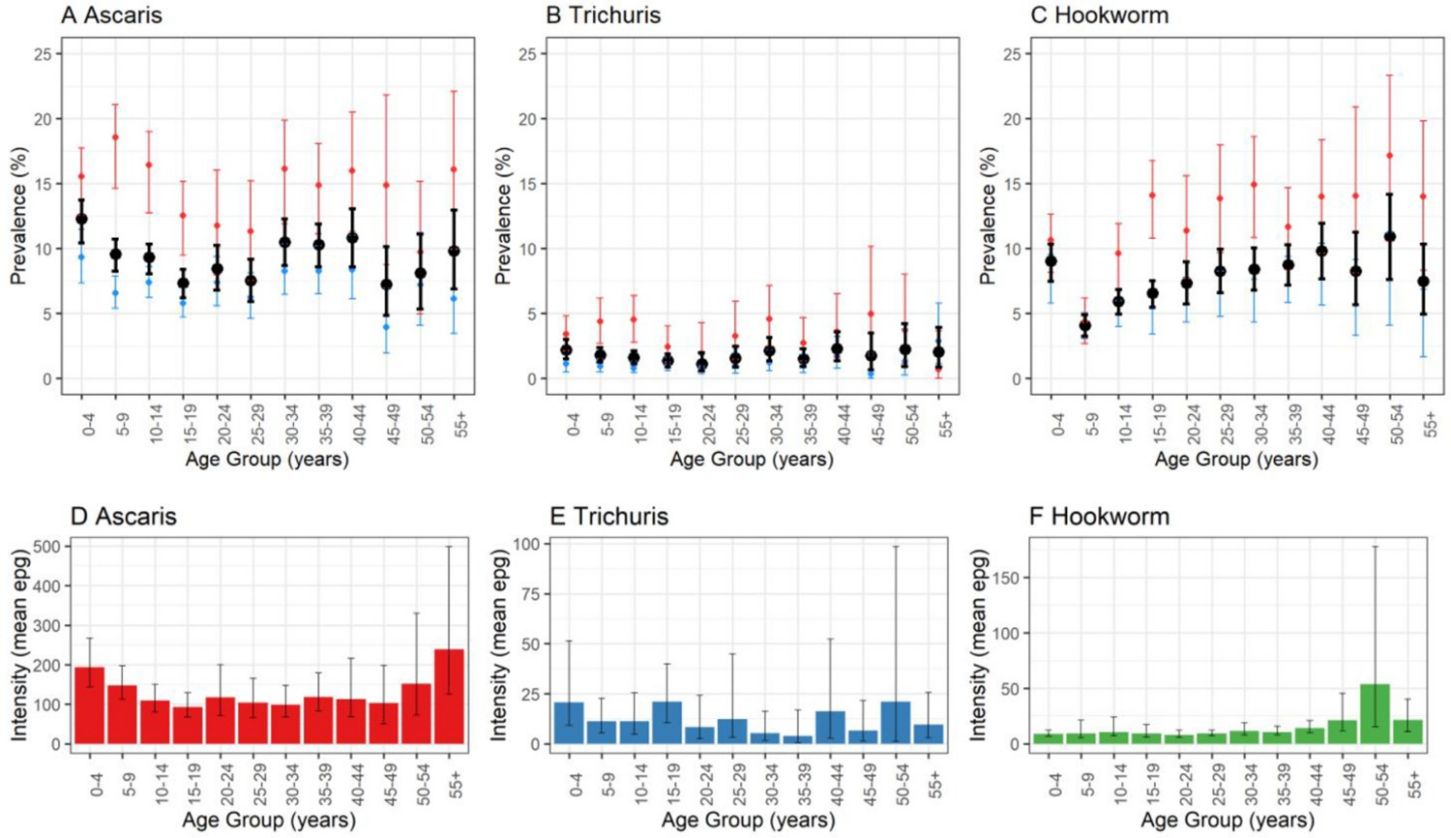
Age prevalence and intensity profiles for STH infections. Age-prevalence estimate in black (combined data), with the baseline mapping age-prevalence estimate in blue and sentinel site baseline estimate in red for *A*. *lumbricoides* (a), *T*. *trichiura* (b) and hookworm (c). Age-intensity profiles for *A*. *lumbricoides* (d), *T trichiura* (e) and hookworm (f), combined data.

Prevalence of hookworm increased with age, although pre-SAC had a noticeably higher prevalence of infection compared to SAC aged (5–14 years), Fig 3C. Hookworm intensity, however, was not higher for pre-SAC compared to SAC, with infection intensity remaining low until late adulthood, Fig 3F. For *A*. *lumbricoides*, and to a lesser extent *T*. *trichiura*, prevalence of infection was also higher in pre-SAC compared to SAC. These age-prevalence profiles were less marked than for hookworm. No statistically significant difference in STH prevalence was observed by sex.

**Fig 4:**
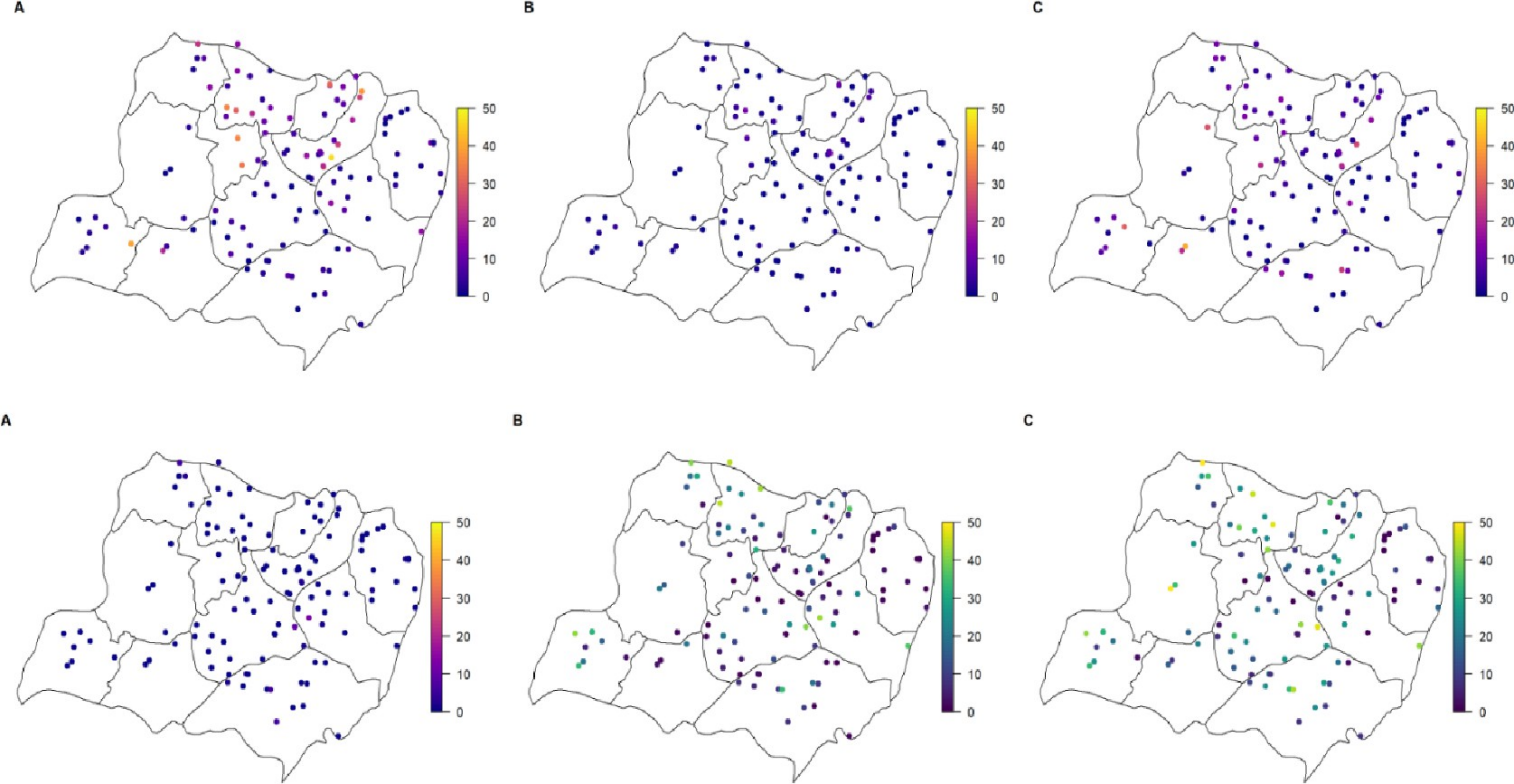
**A–Village level STH prevalence by species, for *A*. *lumbricoides* (a), *T*. *trichiura* (b), and hookworm (c)** and **B—Village level SCH prevalence for S. haematobium (A) and S. mansoni by KK (B), POC-CCA, trace negative (C) and trace positive (D)** This map was created in R software program and the source of the basemap within the package used to generate the map was Global Administrative Areas (2012). GADM database of Global Administrative Areas, version 2.0. [online] URL: www.gadm.org.

Fig 4 shows village-level STH prevalence, S1 and S2 Tables show prevalence and infection intensity summaries for the baseline mapping and sentinel site surveys, respectively, and S3 Fig demonstrates the village-level comparison of prevalence estimates between the two surveys.

**Fig 5:**
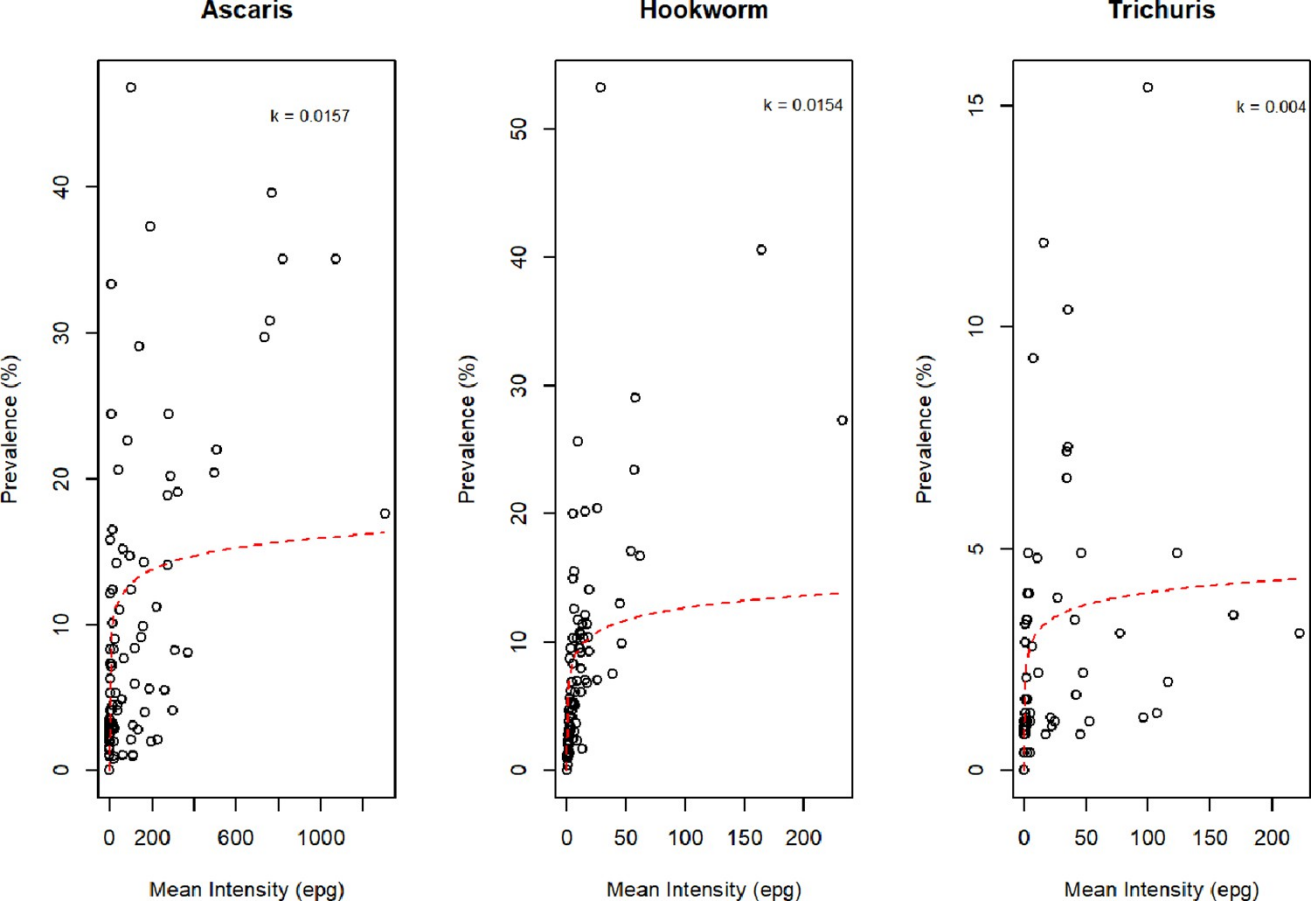
**Village level prevalence and mean intensity of infection for *A*. *lumbricoides* (a), hookworm (b) and *T*. *trichiura* (c).** Each circle represents the village point prevalence estimate and mean epg (including all non-infected individuals). The red dashed line represents the fit of Eq 1, {p = [1-(1+M/k)^-k^]}, that describes the relationship between prevalence (p) and the mean intensity of infection (M) as a function of the level of aggregation of eggs (a proxy for worm burden) in the population (k).

Parasite (egg) aggregation was high for all three STHs, where *k* was estimated across all samples, including the uninfected class. Parasite aggregation (*k*) was estimated to be 0.016 (*A*. *lumbricoides*), 0.015 (hookworm) and 0.004 (*T*. *trichiura*). The prevalence-mean intensity curves, Fig 5, are highly skewed with very low mean epg values for villages with moderate prevalence estimates.

#### Prevalence of SCH infection

Overall prevalence of *S*. *mansoni* infection by KK was 0.85% (CI 0.71–1.01%) and by POC-CCA was 13.31% (CI 12.75–13.88%) when trace results were taken as negative, or 21.55% (CI 20.89–22.21%) trace taken as positive (Fig 4 shows village-level SCH prevalence). Only 11 moderate or heavy intensity infections were observed. Prevalence was marginally higher in males compared to females by POC-CCA trace positive (*χ*^2^ = 4.2, p = 0.04). However, no statistically significant difference was observed for KK or POC-CCA trace negative. Few cases of *S*. *haematobium* infection were observed by urine filtration on haematuria positive individuals only (n = 20, 0.13%). As expected, microhaematuria in urine was higher at 2.77% (CI 2.51–3.05%).

**Fig 6:**
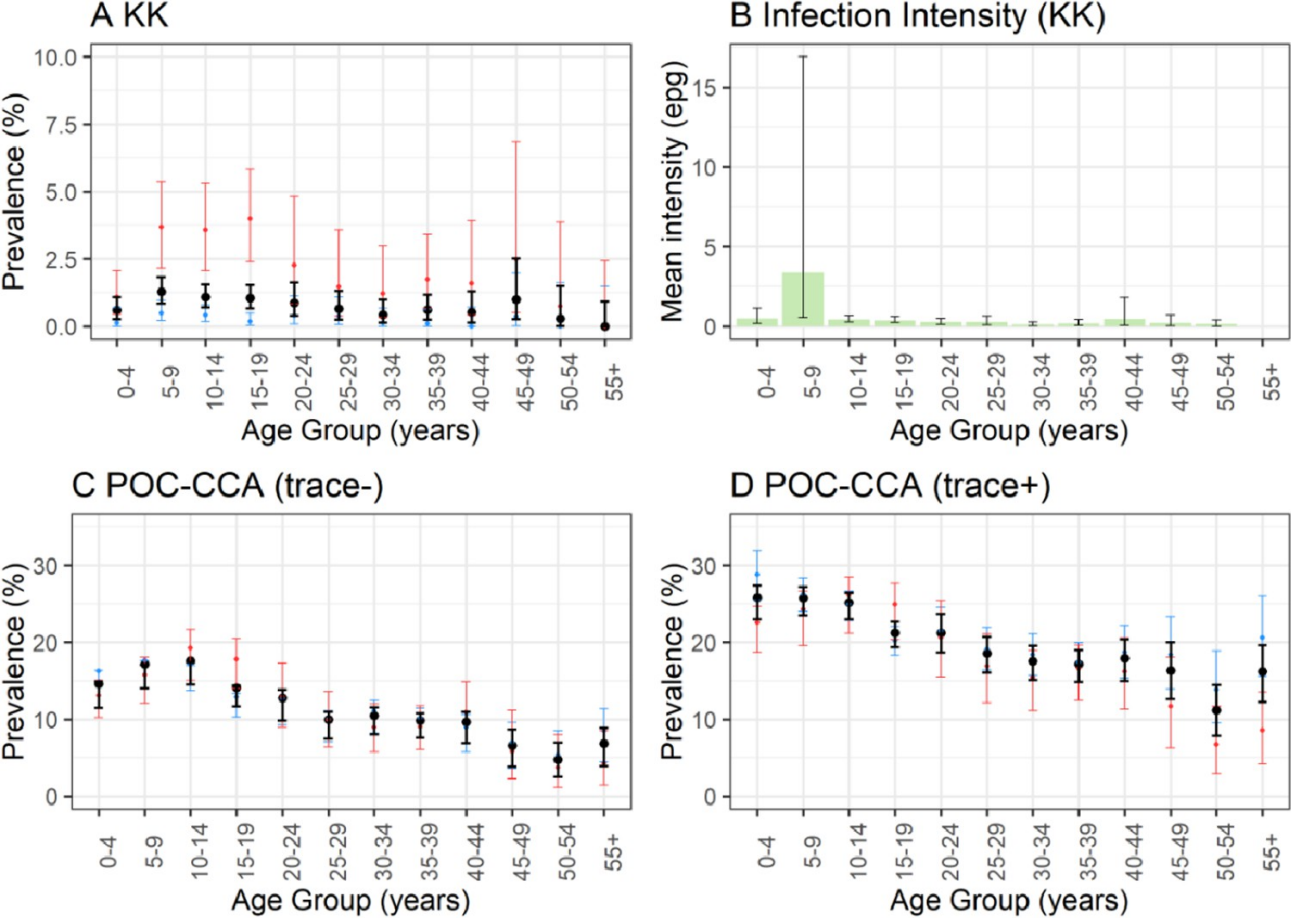
***S*. *mansoni* age-prevalence profiles for diagnostics KK (a), POC-CCA trace negative (c), and POC-CCA trace positive (d).** Age-intensity profile for *S*. *mansoni* infection (b). Note the scale for KK differs from POC-CCA. Age-prevalence estimate in black (combined data), with the baseline mapping age-prevalence estimate in blue and sentinel site baseline estimate in red.

Prevalence of *S*. *mansoni* infection by KK was 0.56% (CI 0.38–0.80%) in Arm 1 and 1.02% (CI 0.83–1.24%) in Arm 2 (*χ*^2^ = 8, p = 0.005). Prevalence by POC-CCA was higher in Arm 1 compared to Arm 2, with trace negative 15.81% in Arm 1 (CI 14.81–16.85%) vs. 11.93% in (CI 11.27–12.62%) in Arm 2 (*χ*^2^ = 41.5, p < 0.001) and trace positive 26.87% in Arm 1 (CI 25.70–28.07%) vs. 18.53% (CI 17.76–19.32%) in Arm 2 (*χ*^2^ = 143, p < 0.001). Parasite prevalence by KK was significantly higher in the sentinel site survey (duplicate KK over two days, total four slides) than the baseline parasitological mapping (duplicate KK on a single day, total two slides) in the SAC and adolescent age groups, Fig 6A. POC-CCA prevalence was consistent between the baseline mapping and sentinel sites. *S*. *mansoni* intensity peaked in the 5-9-year age group, however, this is likely due to one individual’s heavy intensity infection (5,940 epg). When this outlier was removed, the peak for infection remains in the 5–9 age group, albeit at a smaller peak intensity (0.64 mean epg).

## Discussion

### Census

The Geshiyaro census is the first project to use innovative biometric fingerprint technology in the context of NTDs. The rationale for using this technology with a unique identifier, biometric and/or study ID card, is to multi-fold (1) to link individual’s census data with parasitological results and treatment compliance at consecutive MDA rounds and (2) to provide an accurate denominator for treatment coverage, which is often a limitation encountered by NTD programs when estimating PC treatment coverage. This will enable analysis of the impact of random versus systematic (non-compliance to treatment on infection levels as well as evaluation of characteristics of individuals that do (or not) comply to treatment.

There were challenges, however, to registering individuals with unique identifiers. Firstly, there was a degree of reticence to the biometric within some communities despite community sensitization efforts. This was partly because (external) university graduates were used as data collectors (due to the complexity of the technology being used) who were not accepted by the communities. Therefore, an ID card with a unique barcode was used as a secondary method of identification for every participant. Secondly, there was a significant amount of data cleaning required to remove duplicate biometric registrations (where data recorders entered the same person and their fingerprint more than once or sometimes their own fingerprint for other individuals) and household barcodes, which required cross-referencing demographic (name, age, sex, location) data of all household members. As a result of the lessons learned in the pilot census and the logistics required to collect and then clean the data, the census was performed in five (rather than the originally planned 15) districts in Wolaita. Finally, there was a significant amount of time taken to estimate the population denominator even when the data was cleaned. This comprised of four components: the population registered at census (ID or biometric), people declining registration at the census (calculated as the number of declining households in a community multiplied by the average number of people were household), population of potentially missing households (estimated from the number of households censused in Geshiyaro and health bureau estimates for each community), and individuals identified as non-registered during the mapping/sentinel sites/MDA. In summary, a denominator based on contacted households alone would underestimate the true population in a community.

### Baseline parasitology

The Geshiyaro project aims to establish if a combination of expanded CWT, WaSH and BCC interventions can feasibly break transmission of STH and SCH through the existing public health infrastructure of the Federal Ministry of Health [21]. The Wolaita zone was selected by the Ministry for the low (<10%) STH and SCH prevalence found during the national mapping survey because of multiple rounds of historical school-based deworming since 2015 [28]. Despite the relatively small percentage change this requires to reach therefore transmission interruption, when the incidence of new infections in a community is reduced to zero, the effort to achieve this last mile entails multi-pronged interventions, including consistent high MDA coverage and universal community buy-in [29].

Overall, STH prevalence and intensity were found to be low at baseline (9.5% *A*. *lumbricoides*, 1.8% *T*. *trichiura*, and 7.2% hookworm) but there was substantial heterogeneity between and within districts. Such patterns of heterogeneity have been observed in other STH control trials, for example in the TUMIKIA and DeWorm3 [11, 13]. There was also difference in prevalence seen between arms. Arm 1 had a higher STH prevalence, largely focused on the northern half of Wolaita, which is more temperate than the south and was found to have lower WaSH coverage. The starting prevalence and baseline WaSH will be taken into account when assessing the impact of the interventions at the end of survey in 2025.

The prevalence-mean intensity curves are highly skewed with very low mean epg values for villages with moderate prevalence estimates, reflecting the impact of past MDA treatment. This pattern equates to most people being uninfected or harbouring a low intensity infection and a small number of people with moderate intensity infections that facilitate continued parasite transmission.

Prevalence of *S*. *haematobium* was found to be low at 0.1% by urine filtration, however, filtration was carried out on haematuria positives only and there is a limitation in accuracy of detecting ultra-light infections. More sensitive diagnostics will be needed at the end of Geshiyaro to reliably determine interruption of transmission [30]. Prevalence of *S*. *mansoni* was 0.8% by KK but increased to 13.3% and 21.5%, when assessed by POC-CCA trace negative and positive, respectively. POC-CCA detects *S*. *mansoni* antigens in urine and is more sensitive at picking up low-intensity infections in areas of low-to-moderate prevalence [31,32]. The estimate of 1% prevalence by duplicate KK slide readings corresponds to a range of 5% to 25% when POC-CCA trace readings are considered positive [33, 34]. The decision on whether to treat traces as positive remains an ongoing debate, particularly in low prevalence settings where there is a need to balance the benefit of identifying very low intensity infections with the risk of false positives [35,36].

A difference was also observed between the baseline mapping and the sentinel site survey. This can primarily be explained as double the number of KK were used in the sentinel sites compared to the mapping (four vs. two slides). An increased number of slides were used pre-emptively to ensure infection was identified in the final years of the project when there was anticipated to be low or no infection. As this study was aimed to measure transmission break it is crucial to have highly sensitive diagnostics such as quantitative polymerase chain reaction (qPCR) used at the endline mapping to determine if interruption of transmission has been achieved in addition to KK [37]. A subset of sentinel site stool samples have been stored each year for analysis to quantify the relationship between *S*. *mansoni* and STH intensity of infection by KK and qPCR and how that changes over the course of the intervention [38].

Both STH and SCH infections were found to be highly aggregated as is typical for all helminth infections of humans, meaning few individuals harboured a disproportionately high number of eggs (a proxy for worms), as indicated by low negative binomial *k* values for each STH species, while most people harboured no or light infections [39]. It is known that aggregation of worms increases as prevalence decreases, with *k* values displaying a corresponding decrease. This is to be expected given the multiple rounds of PZQ and ALB given through school-based deworming and one district (Sodo Zuria) receiving ALB via CWT against lymphatic filariasis [40]. High parasite aggregation, in combination with significant geographic heterogeneity in prevalence at the village level, will be influenced by non-adherence to treatment in a fraction of the population. Individuals in a community who have never received deworming treatment, particularly when basic or improved sanitation facilities are not present, may maintain a reservoir of infection within the community, hampering potential progress in elimination through MDA. Access to basic or safely managed sanitation facilities was limited (<20%) in all five woredas censused, with most households having unimproved latrine facilities. Therefore, even if the entire population was compliant to treatment there would be reinfection by a contaminated environment. Given that the parasites cannot survive indefinitely if the population can ceases putting the eggs/larvae back into the environment the cycle should cease.

The Ethiopian National Control Program has made significant gains in reducing overall STH infection since the programme’s conception in 2015 [28, 41]. The impact of previous school-based MDA are observed through the flat age-prevalence and intensity profiles for both *A*. *lumbricoides* and *T*. *trichiura*, which typically exhibit a distinct peak in SAC in endemic settings prior to repeated MDA administration [11,42]. The typical increase in hookworm prevalence and intensity of infection with increasing age was also observed. There was an unanticipated, elevated hookworm (and *A*. *lumbricoides*) prevalence observed in pre-SAC at baseline, despite pre-SAC being dewormed through the Nutrition Program that may indicate poor coverage in that age group. These findings suggest that expansion of deworming to these age groups could facilitate the reduction of prevalence below the threshold that is needed for transmission interruption.

One key finding of the baseline parasitological mapping results was the extent of heterogeneity in infection observed. As national control programmes successfully lower levels of overall STH and SCH prevalence, the evolution of national goals from elimination as a public health problem to interrupting transmission will mean a more tailored approach to PC. At present, national treatment decisions are made at the district level, which may result in the over or undertreatment of segments of the population. Modification of treatment strategies on a sub-district (or kebele) level could reduce misallocation of treatment by focusing control efforts on persistent hotspots of infection. Targeting treatment to both those predisposed to high infection and to villages with higher-than-average infection levels will need to be considered as overall prevalence levels fall and therefore use drug supplies optimally.

### Limitations

Overall prevalence of STH was higher in the sentinel site parasitological survey compared to baseline mapping. This is likely due to several reasons. There was a time gap between the baseline mapping in December 2018 and the completion of all sentinel site baseline surveys by March 2020 (in all districts apart from Bolosso Sore). Most significantly, the diagnostic methods differed between surveys with four KK slides in the sentinel sites and two slides in the mapping. Previous studies have demonstrated improved diagnostic sensitivity, particularly in low prevalence settings, as the number of slides increases [43].

The districts that border Lake Abaya did not exhibit higher prevalence of SCH infection than districts further from the lake as anticipated. This may be due to the random selection and therefore low number of mapping sites near the water. A hybrid sampling strategy that combined both random and purposive sampling could be a more accurate strategy going forward as countries look to assess the impact of multiple rounds of treatment. Alternatively, information and education messages around risk of SCH may have played a role as households in Abela Abaya self-reported less exposure to surface water through washing clothes or bathing, despite proximity to a large lake, than the four other census districts (56.8% compared to 65.1–82.2%).

The impact of the COVID-19 pandemic will be difficult to measure, however, NTD programming including MDA and surveys were paused in 2020. As a result, the endline survey for Geshiyaro was moved from 2023 to 2025. Conversely, COVID-19 prevention methods such as the installation of hand washing stations and community information on hygiene may indirectly benefit STH (and to a less extent SCH).

## Conclusion

The Geshiyaro parasitological survey sampled 40% of the villages across all districts in Wolaita, providing a highly granular spatial picture of helminth infection prevalence across the zone. Furthermore, all age groups were surveyed, expanding from the more common strategy of SAC-centric measures. This provides important insights into age-prevalence and age-intensity patterns across the whole population following multiple rounds of STH and SCH school-based treatment. Looking prospectively to the endline survey this dataset provides an excellent base on which geospatial techniques could be applied to develop a robust impact assessment protocol to re-evaluate SCH and STH following multiple years of MDA [44–45]. Finally, the Geshiyaro study is the only large-scale survey to have collected unique individual identifiers through biometric registration to follow compliance to MDA treatment and access to WaSH over time, and the concomitant impact on SCH and STH infection levels. This will enable high detail, individually linked, longitudinal monitoring of the enrolled population (about 600,000 participants across the five biometrically monitored sites), making it one of the largest longitudinal studies of STH and SCH intervention control conducted to date.

## Supporting information

S1 Fig Proportion of men/women by parasitology age group, mapping, and sentinel sites. The age-sex stratification of participants in the mapping and sentinel site surveys.
(TIF)

S2 Fig Census age pyramids by district. Proportion of individuals enrolled across the five censused districts by age group in the Geshiyaro project, in comparison to the World Bank population estimates, young men and women in their early twenties and pre-school age children were relatively under sampled.
(TIF)

S3 Fig Scatter plot of mapping vs. sentinel site prevalence for any STH, Ascaris, Trichuris, and hookworm. Baseline mapping occurred in November-December 2018. Phase I sentinel sites were conducted in January 2019 (villages shown in blue), with the following 26 Phase II sites conducted in February-March 2020. The figure demonstrates the village-level comparison of prevalence estimates between the mapping and sentinel site surveys.
(TIF)

S1 Table Prevalence and intensity of infection, baseline mapping data. S1 Table shows prevalence and infection intensity summaries for STH and SCH (by species) from the baseline mapping conducted between November 2018 and January 2019.
(XLSX)

S2 Table Prevalence and infection intensity summaries for the sentinel site surveys. **S2** Table shows prevalence and infection intensity summaries for STH and SCH (by species) from the baseline sentinel site survey conducted in four sites in Bolosso Sore district in January 2019 and the remaining 26 sites across Wolaita between February and March 2019.
(XLSX)
