# Supplementary figures and images for: Baseline soil-transmitted helminth and schistosome infection in the Geshiyaro project, Ethiopia: A unique transmission interruption project using biometric fingerprinting for longitudinal individual analysis
Source: PLoS Negl Trop Dis. 2023 Oct 18;17(10):e0011589. doi: 10.1371/journal.pntd.0011589 (PMC10615263; doi:10.1371/journal.pntd.0011589)

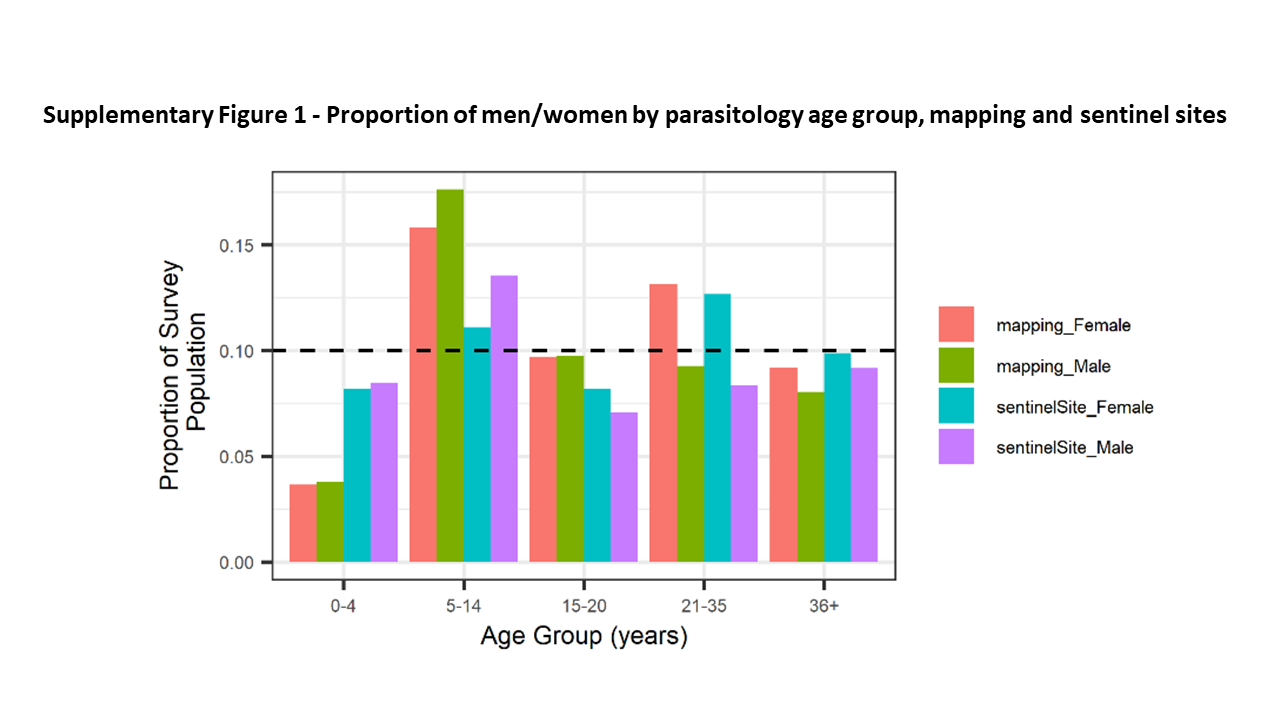

Supplement: S1 Fig — The age-sex stratification of participants in the mapping and sentinel site surveys. (TIF) [file pntd.0011589.s001.tif]

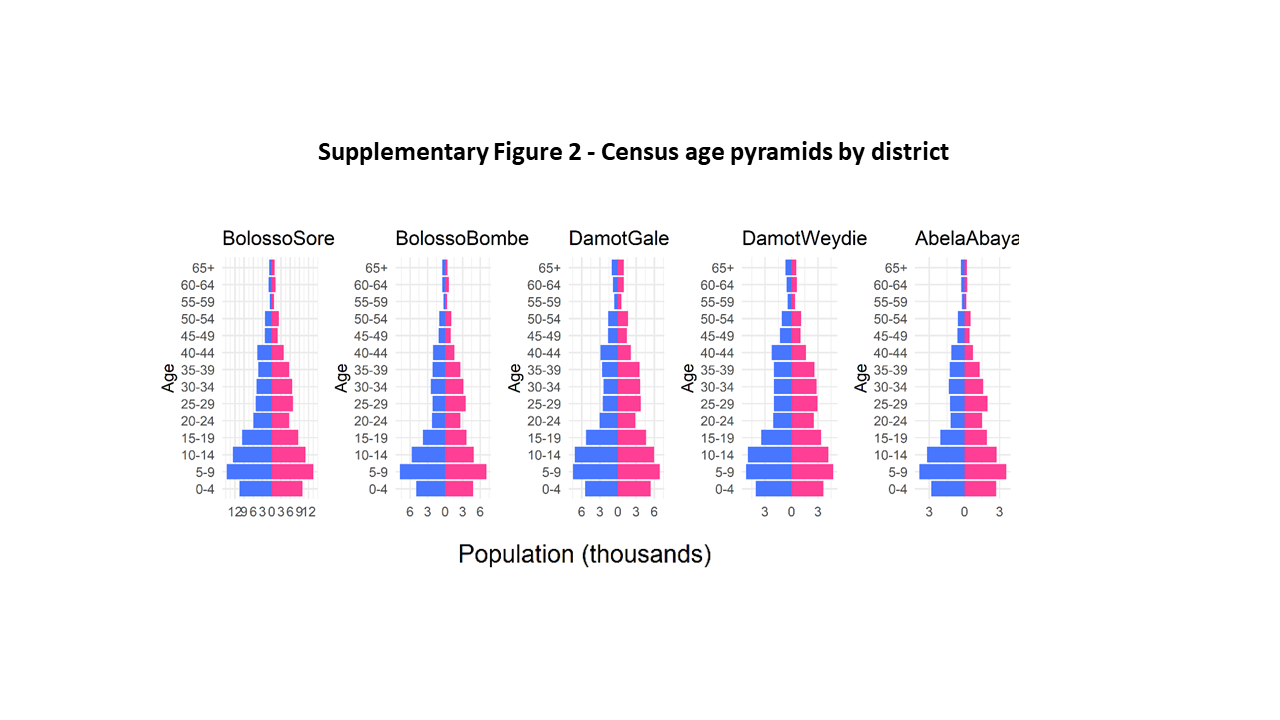

Supplement: S2 Fig — Proportion of individuals enrolled across the five censused districts by age group in the Geshiyaro project, in comparison to the World Bank population estimates, young men and women in their early twenties and pre-school age children were relatively under sampled. (TIF) [file pntd.0011589.s002.tif]

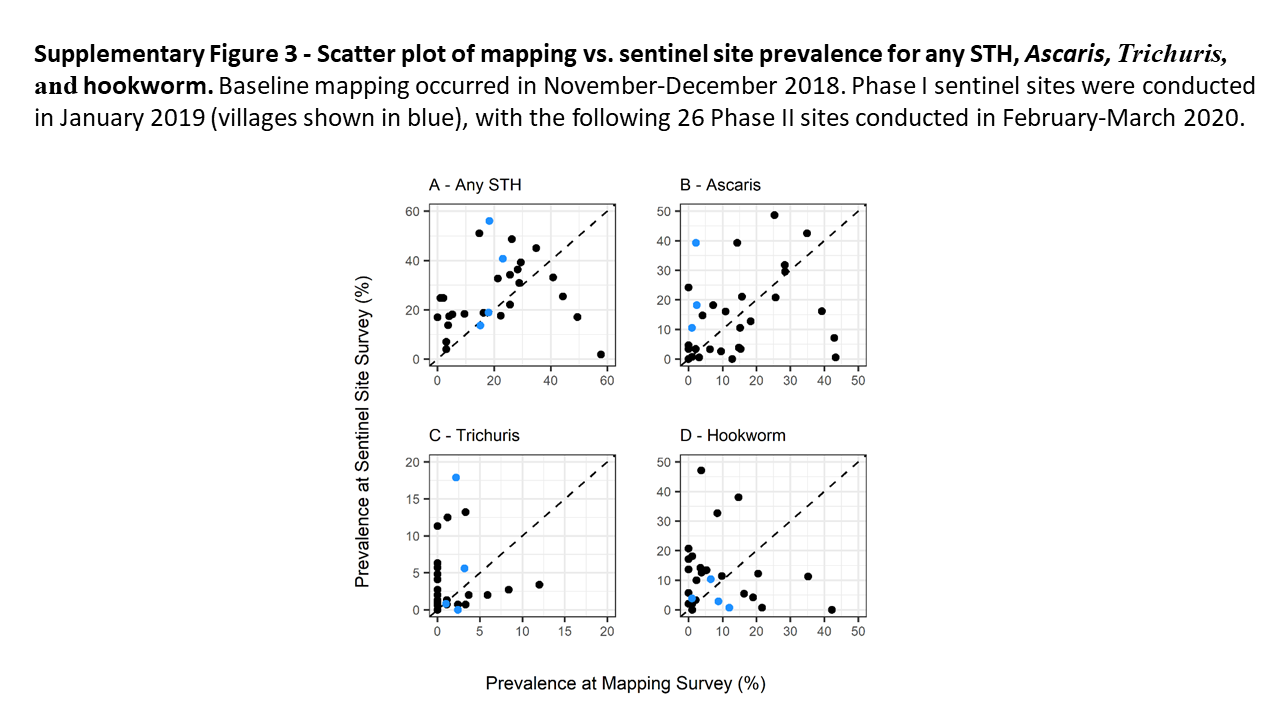

Supplement: S3 Fig — Baseline mapping occurred in November-December 2018. Phase I sentinel sites were conducted in January 2019 (villages shown in blue), with the following 26 Phase II sites conducted in February-March 2020. The figure demonstrates the village-level comparison of prevalence estimates between the mapping and sentinel site surveys. (TIF) [file pntd.0011589.s003.tif]
